# Supplementary figures and images for: Streptococcus pneumoniae drives specific and lasting Natural Killer cell memory
Source: PLoS Pathog. 2023 Jul 24;19(7):e1011159. doi: 10.1371/journal.ppat.1011159 (PMC10399893; doi:10.1371/journal.ppat.1011159)

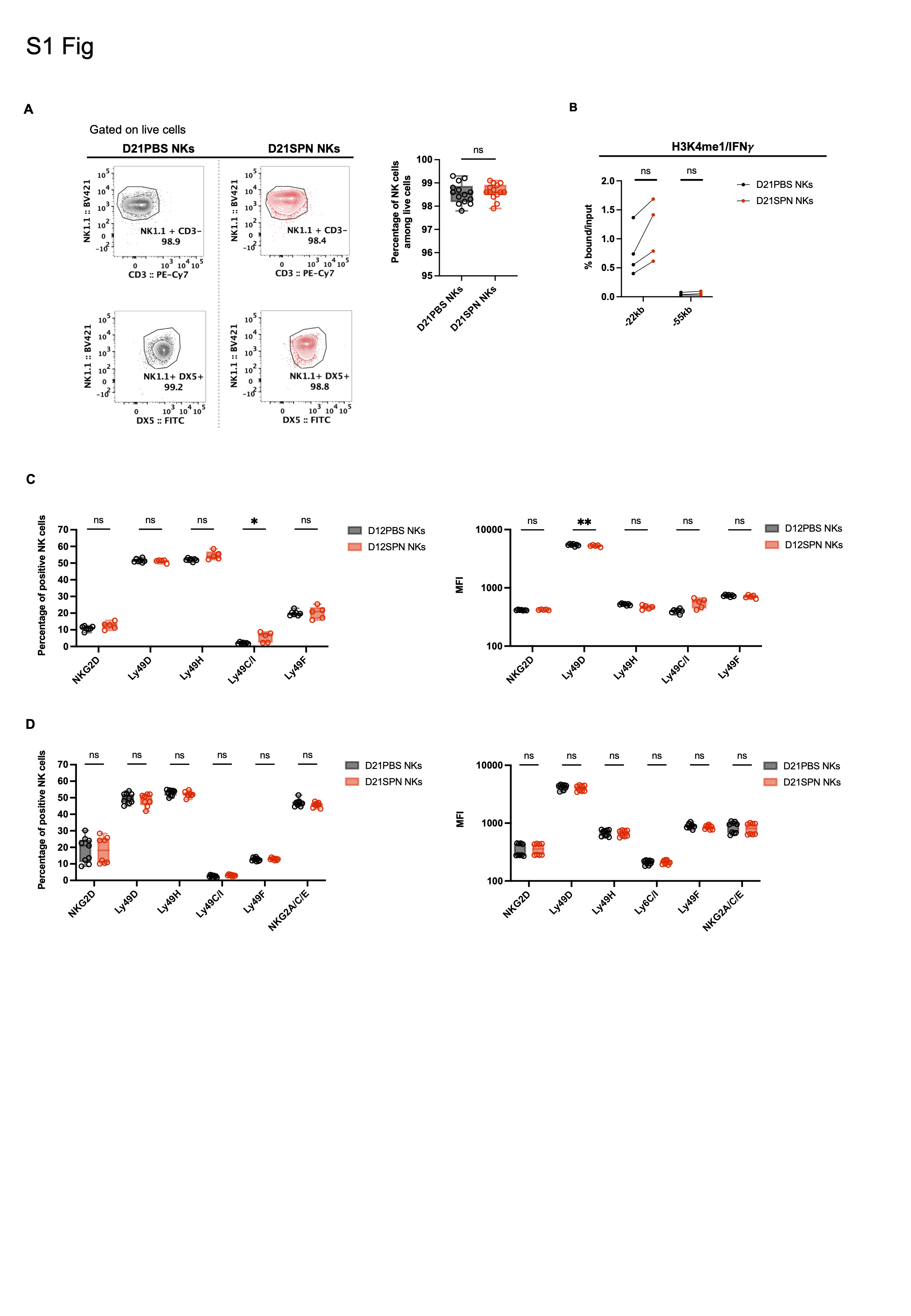

Supplement: S1 Fig — (A) Representative contour plots for NK cell purity of D21PBS (black symbols) and D21SPN (red symbols) samples based on NK1.1+ CD3- or NK1.1+ DX5+ markers (left panel). Percentage of NK cells among live cells (purity of cells, right panel). Box plots where each dot represents a pool of mice from one experiment, lines are the median, error bars show min to max. Data are representative of more than three repeats with n ≥4 pooled mice/group and n ≥ 3 experimental replicates/group. (B) Mouse infections are carried out as in the scheme in Fig 1A. NK cells were isolated from spleens of mice previously infected with S. pneumoniae (red bars, D21SPN NKs) or not (black bars, D21PBS NKs). Highly purified NK cells were fixed, and chromatin was extracted and sheared. ChIP for H3K4me1 were performed and resulting positive fractions of the chromatin were amplified using PCR for the indicated targets. Enrichment percentage for H3K4me1 pull-down on regions upstream the ifng gene in NK cells from D21PBS and D21SPN mice. Data are representative of four experiments with n ≥ 4 mice/group. (C-D) Splenocytes were harvested from mice 12 days (C) or 21 days (D) after they were intranasally injected with either PBS (black symbols) or sub-lethal dose of S. pneumoniae (SPN, red symbols, 5x10^5^ CFU) for two consecutive days. NK cell expression of several NK cell receptors in percentages (left panel) and intensity of expression (MFI, right panel). Box plots where each dot represents an individual mouse, lines are the median, error bars show min to max. Data are pooled from one (C) or two (D) repeats with n ≥ 4 mice/group. ns, not significant. * p < 0.05 and ** p <0.01. Mann-Whitney (A,B) and 2way ANOVA (C,D) tests for statistical significance. (TIFF) [file ppat.1011159.s001.tiff]

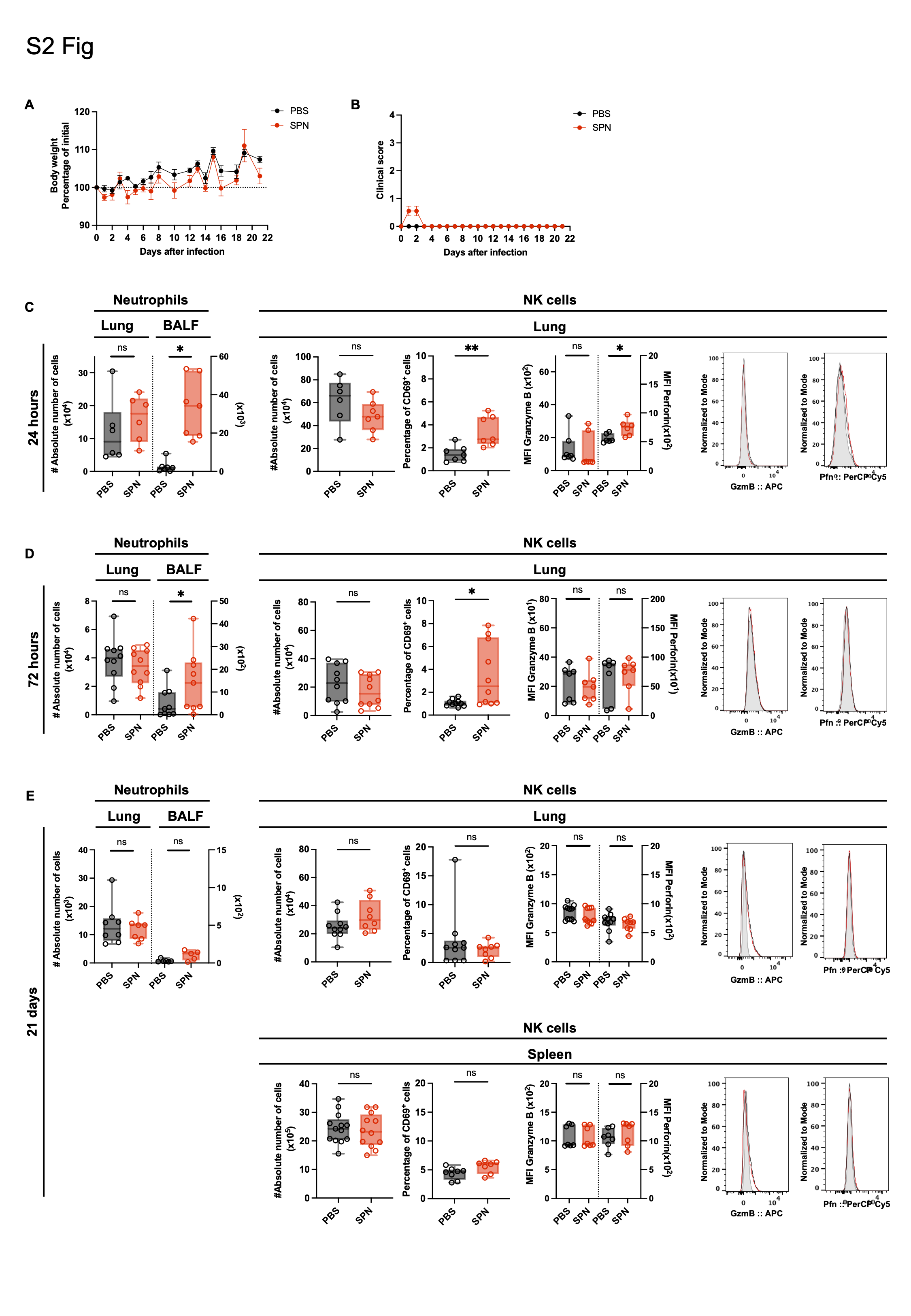

Supplement: S2 Fig — Weight (A) and clinical score (B) following the infection scheme from Fig 2A. Dots represent the mean and error bars are the standard error of the mean (SEM). Data are pooled from two repeats with n = 4 mice/group. (C-E) Organs were collected at 24h (C), 72h (D) and 21 days post-infection (E). Absolute numbers of neutrophils (CD11b+ Ly6G+) in the lungs and bronchio-alveolar lavage fluid (BALF) (left panel). Absolute numbers of NK cells (NK1.1+ CD3-), percentage of CD69+ NK cells, intensity of Granzyme B and Perforin expression (MFI) and representative overlay histogram of Granzyme B and Perforin staining in NK cells (right panel). Box plots where each dot represents an individual mouse (black dots for uninfected mice, red dots for infected mice), lines are the median, error bar show min to max. Grey histogram represents isotype control. Data are pooled from two or three repeats with n ≥ 3 mice/group. ns, not significant. * p < 0.05, ** p < 0.01. Mann-Whitney test for statistical significance. (TIFF) [file ppat.1011159.s002.tiff]

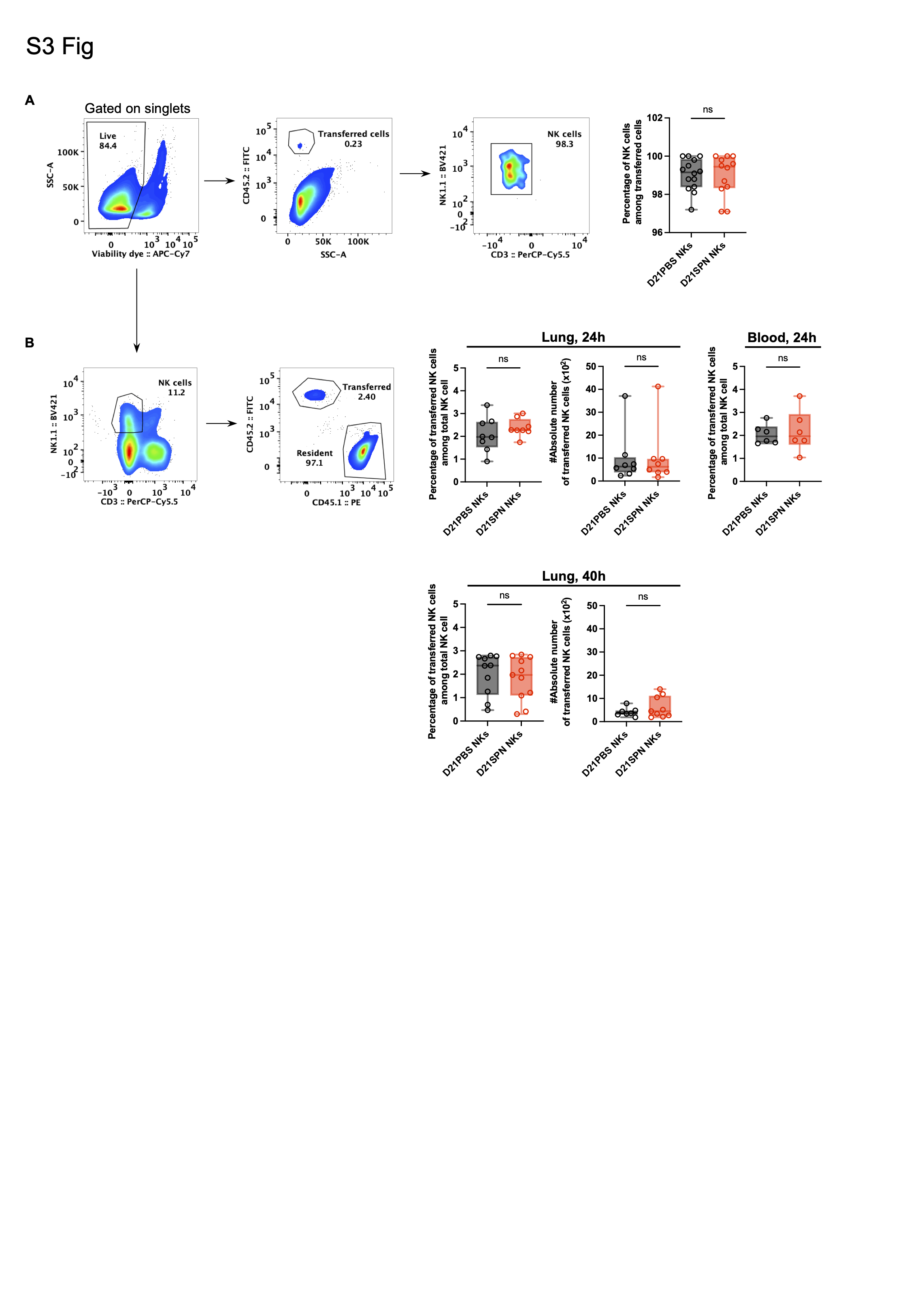

Supplement: S3 Fig — (A-B) Mouse infections are carried out as in the scheme in Fig 3A. Congenic mice are used to distinguish donor NK cells (CD45.2+) from recipient NK cells (CD45.1+). (A) Representative gating strategy to measure purity of transferred D21PBS NKs (black symbols) or D21SPN NKs (red symbols) in the lungs of recipient mice at 24 hours post-infection. Purity represents the percentage of NK cells among CD45.2+ transferred cells. Box plots where each dot represents an individual recipient mouse, lines are the median, error bar show min to max. Data are representative of three repeats with n ≥ 4 mice/group. (B) Representative gating strategy to detect transferred NK cells. Percentages and numbers of CD45.2+ transferred NK cells in the lungs and blood of CD45.1+ recipient mice at 24h and 40h post-infection. Box plots where each dot represents an individual recipient mouse, lines are the median, error bar show min to max. Data are pooled from at least two repeats with n ≥ 3 mice/group. ns, not significant. Mann-Whitney test for statistical significance. (TIFF) [file ppat.1011159.s003.tiff]

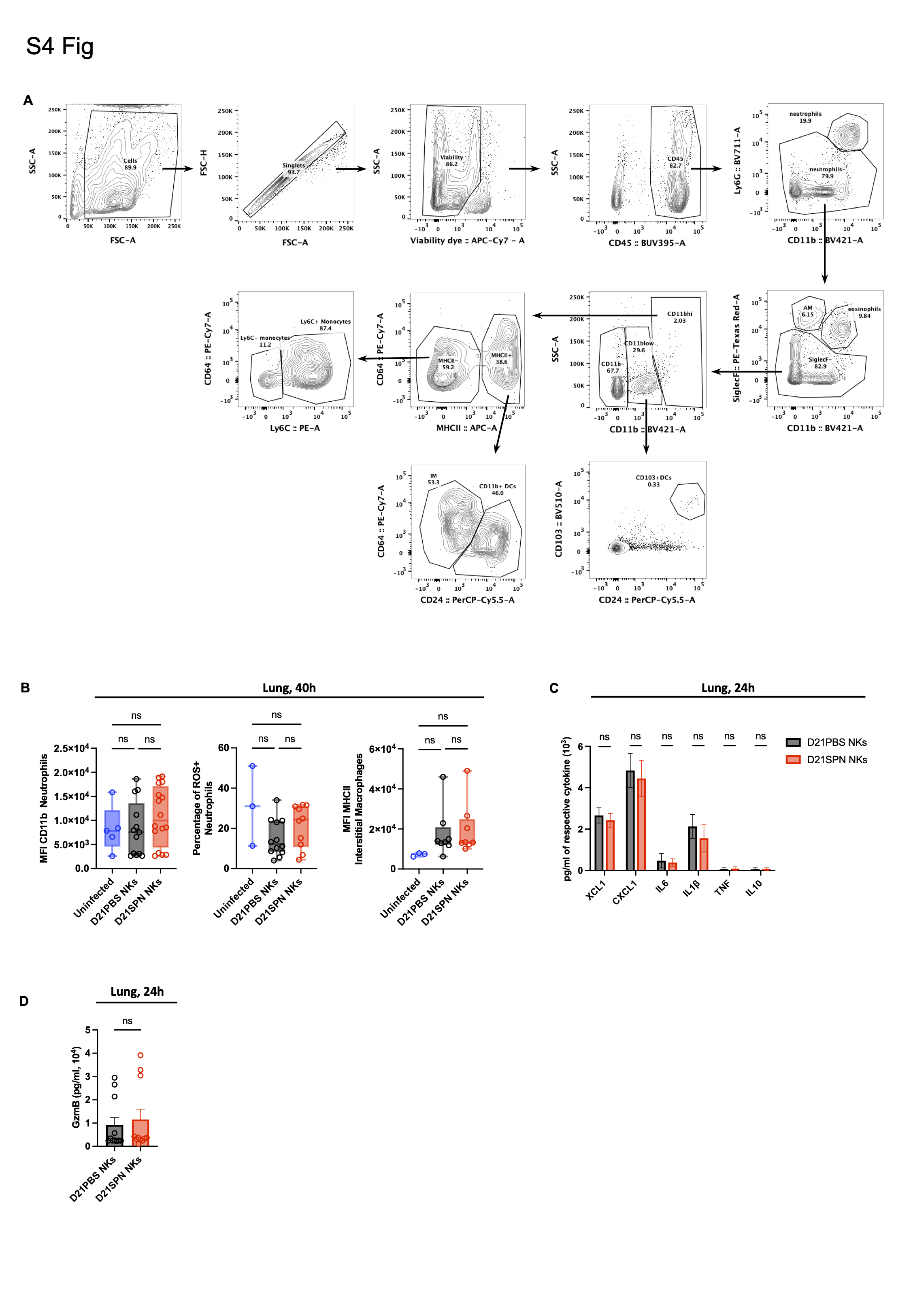

Supplement: S4 Fig — Mouse infections are carried out as in the scheme in Fig 3A. Organs were collected at 24h and 40h post-infection for flow cytometry analysis and ELISA assays. (A) Gating strategy used to identify myeloid-cell subsets in the lungs at 24h post-infection, adapted from [58]. After the exclusion of debris, doublets and dead cells, immune cells were identified by CD45 staining. Neutrophils, alveolar macrophages and eosinophils are defined with the following specific markers respectively: Ly6G+ CD11b+, SiglecF+ CD11b-, SiglecF+ CD11b+. Gating on CD11bhigh was used to distinguish myeloid cells from lymphoid cells with the exception of CD103+ dendritic cells that are defined as CD11blow CD103+ CD24+ cells. In CD11bhigh subset, gating on MHCII+ cells allow to identify interstitial macrophages as MHCII+ CD64+ CD24- cells and CD11b+ dendritic cells as MHCII+ CD64- CD24+ cells. On the contrary, CD11bhigh MHCII- cells are monocytes/immature macrophages that can have different maturation states based on Ly6C marker. (B) Cellular activation analysis in the lungs at 40h post-infection. Intensity of CD11b expression in neutrophils (MFI), percentage of ROS+ neutrophils and intensity of MHCII expression in interstitial macrophages (MFI). Box plots with each dot representing individual mice (blue dots for uninfected mice, black dots for mice having received D21PBS NKs, red dots for mice having received D21SPN NKs), lines are the median, error bar show min to max. Data are pooled from two repeats with n ≥ 1 mice/group. (C) ELISA assays of lung supernatants from infected mice having received either D21PBS NKs (black) or D21SPN NKs (red) at 24h post-infection. Bars are the mean of at least 3 experiments with n ≥ 3 mice/group, error bars are the standard error of the mean (SEM). (D) Granzyme B ELISA assays of lung supernatants from infected mice having received either D21PBS NKs (black) or D21SPN NKs (red) at 24h post-infection. Bars are the mean of at least 3 experiments with n ≥ 3 mice/gr [file ppat.1011159.s004.tiff]

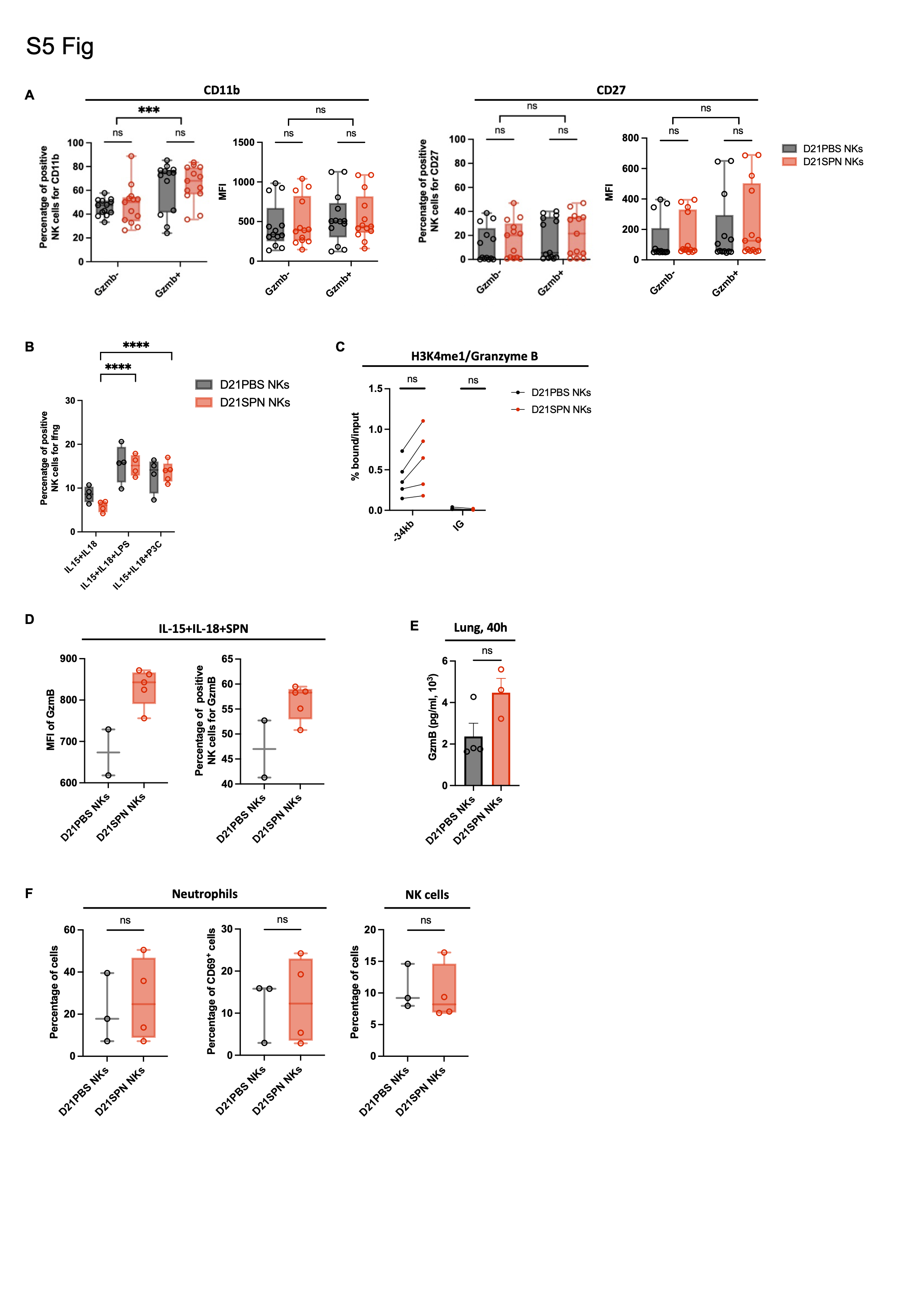

Supplement: S5 Fig — (A) NK cells were stimulated in vitro with cytokines and formaldehyde inactivated SPN (MOI 20) for 24 hours. Percentage and intensity (MFI) of CD11b in NK cells (left panel), percentage and intensity (MFI) of CD27 in NK cells (right panel) in both Granzyme B+ and Granzyme B- cells. D21PBS NK cells and D21SPN NK cells are purified and pooled from n ≥ 4 mice/group and incubated in n ≥ 3 experimental replicates/group. Box plots where each dot represents an experimental replicate (black dots for D21PBS NK cells, red dots for D21SPN NKs cells), lines are median, error bar show min to max. Data are representative of three experiments. (B-C) C57BL/6 mice were intranasally injected with either PBS (black symbols) or sub-lethal dose of S. pneumoniae (SPN, red symbols, 5x10^5^ CFU) for two consecutive days. After 21 days, NK cells were highly purified from spleens of D21PBS or D21SPN mice (98% of purity) and stimulated in vitro with cytokines (IL-15 at 2 ng/ml, IL-18 at 1,5 ng/ml, IL-12 at 1,25 ng/ml) and either formaldehyde inactivated SPN (MOI 20), Lipopolysaccharide (LPS) or the synthetic lipopeptide Pam3CSK4 (P3C) for 24 hours. (B) NK cells were stimulated in vitro with cytokines, Lipopolysaccharide (LPS) and the synthetic lipopeptide Pam3CSK4 (P3C) for 24h. Percentage of IFNγ+ NK cells. D21PBS NK cells and D21SPN NK cells are purified and pooled from n ≥ 3 mice/group and incubated in n ≥ 2 experimental replicates/group. Box plots where each dot represents an experimental replicate (black dots for D21PBS NK cells, red dots for D21SPN NKs cells), lines are median, error bar show min to max. Data are representative of two experiments. (C) Mouse infections are carried out as in the scheme in Fig 1A. NK cells were isolated from spleens of mice previously infected with S. pneumoniae (red bars, D21SPN NKs) or not (black bars, D21PBS NKs). Highly purified NK cells were fixed, and chromatin was extracted and sheared. ChIP for H3K4me1 were performed and resulting positive fractions [file ppat.1011159.s005.tiff]
