# Supplementary material for: Streptococcus pneumoniae drives specific and lasting Natural Killer cell memory
Source: PLoS Pathog. 2023 Jul 24;19(7):e1011159. doi: 10.1371/journal.ppat.1011159 (PMC10399893; doi:10.1371/journal.ppat.1011159)
Supplement: Attachment — Submitted filename: Reviews PPathogens_NKcells_FINAL.pdf [file ppat.1011159.s006.pdf]

Reviewer #1

1. It is unclear whether lung NK cells from 21d post-infection respond similarly to in vitro stimulation as the spleen NK cells. For example, do they produce IFN-gamma and granzyme B upon stimulation with IL-15/IL-18/SPN?
We agree on the interest of this point. The main reason we use NK cells from spleen is because the spleen is the largest reservoir of NK cells in mice. Technically, a much higher number of mice is needed to purify enough NK cells from lungs. In addition, in vitro experiments require many more NK cells than in vivo transfers. Therefore, technically we have chosen to answer this point by performing in vivo protection experiments. In the revised version of the manuscript, we have now added an experiment where we have transferred purified Memory or Naïve NK cells from lung (rather than spleen as in the original manuscript) to recipient mice in vivo (see Figure 3F). Interestingly, these results show that Memory NK cells from lung have a similar protective effect than those from spleen and contribute to reduce the number of CFUs in recipient mice. This data is now included in Figure 3F.

2. Are there any transient NK cell responses (including IFN-gamma, granzyme B) in spleen NK cells at 24-72 hpi like there are in the lung (in Fig 2B-C)? This would relate to the infection-associated phenotype in spleen NK cells, which are used for the adoptive transfers demonstrating memory NK cell protection.
We present below the data for NK cell responses in spleen following sub-lethal infections (Figure R1). We do not observe any transient response in NK cells percentage, number or activation in the spleen following sub-lethal infection, consistent with the fact that we also do not detect CFUs in this organ. These data are shown below but will not be included in the manuscript as it is all negative data. We hypothesize that NK cells enter in contact with SPN in the lung and only later circulate to other organs such as the spleen.

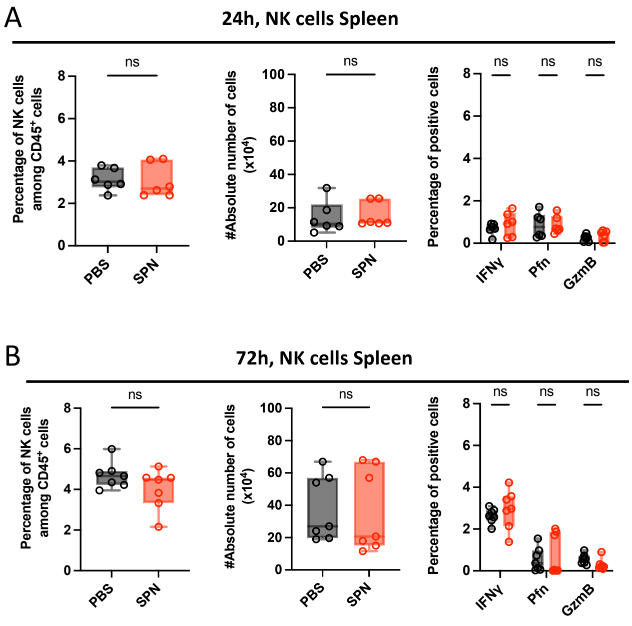

Figure R1: Organs were collected at 24h (**A**), 72h (**B**). Percentage of NK cells (NK1.1$^+$ CD3$^-$) among CD45$^+$ cells, percentage of IFNγ^+, Perforin$^+$, Granzyme B$^+$ NK cells in spleen (**right panel**). Box plots where each dot represents an individual mouse (black dots for uninfected mice, red dots for infected mice), lines are the median, error bar show min to max. Data are pooled from two with n ≥ 3 mice/group. ns, not significant. Mann-Whitney test for single comparisons and 2way ANOVA test for multiple comparisons.

Reviewer #2:

1. The study contains plenty of high quality data, derived from well-powered experimental analyses. However, a couple of important conclusions are reached based on data trends that don't reach statistical significance. The two sections where histone modification were assessed both suggest interesting (and potentially functionally important) changes that may be driven by infection. However, in both cases, the sample size for analysis is small (n=4 and n=5), which prevents firm conclusions from being drawn. I would suggest the authors attempt an extra few replicates, to pin down whether the mechanisms they describe are driven by epigenetic modifications in ifng and gzmb.
   We agree with the reviewer, but technically we do not believe statistical significance will be reached without sacrificing a very large number of animals. Indeed, for these experiments, every replicate is performed by pooling several mice to obtain enough NK cells and enough DNA to do ChIP-qPCR. One extra repeat of this experiment would imply the sacrifice of at least 12 mice and we are not certain about how many repeats would be needed to reach significance. We believe that the fact that we are looking at bulk NK cells, while expecting that the memory cells are only a sub population, contributes to the lack of significance in this experiment. However, we believe the consistent trend suggests long-term chromatin remodeling. Once the subpopulation of memory NK cells is characterized, we will be able to characterize chromatin features in a more convincing manner.

2. Similarly, the differences in mouse survival described in Figure 3C are based on experiments with n=4 per group. This is a very small number for a survival experiment and I would have more confidence in the conclusions reached, if the results were reproduced in a larger sample size.
   We have added one more replicate to this experiment, which is now performed on 24 mice. This has reduced the p-value from 0.069 to 0.060 and is included in a new figure 2. Considering the cruelty of this experiment and the elevated number of mice (two groups of donor mice and two groups of recipient mice), we believe that the current data is sufficient to show that there is a protective phenotype, especially given that significance is achieved when measuring CFU numbers in different organs.

3. The adoptive transfer experiments performed with the ifngr KO mice are nicely conceived, demonstrating that IFNg is not the basis of the protective mechanism at play in NK cell memory of pneumococcal infection. I found the experiments with the perforin KO less convincing. Why were the prf1 KO mice used as recipients in these experiments? Transfer of prf1 KO NK cells with a memory phenotype into WT mice might have been used to demonstrate that perforin production by memory cells was the basis of protection. Transfer into the prf1 KO leaves open the possibility that perforin production by non-memory cells might contribute to protection against pneumococcal infection. What was the trajectory/outcome of infection in the prf1 KO animals? Do they experience worse outcomes or harbour higher bacterial burdens than WT?

To begin by answering the last questions, Perforin KO animals responded similarly to WT mice. Perforin KO NK cells could still acquire memory properties, as measured by increased levels of GzmB following stimulation in vitro, and also in lung supernatants following infection (see Figure S5B and S5C).

For the first part of the question, when NK cells from Perforin KO animals are transferred to WT mice, the protective phenotype is maintained (see below Figure R2). This therefore suggests that Perforin from endogenous immune cells was sufficient for transferred NK cells to perform memory. In contrast, when the experiment is done with KO cells into KO animals, the complete absence of Perforin prevents protective functions. We hypothesize that the main actor is GzmB, which requires Perforin for full activity, and the optimal experiment would have been done with GzmB KO animals, which cannot be easily obtained. Altogether, we agree with the reviewer and want to make sure a clear message is included in the manuscript. We have changed the wording for the conclusion of this section to read “our data suggests that memory NK cell protection from lethal infection is abolished in the absence of Perforin”.

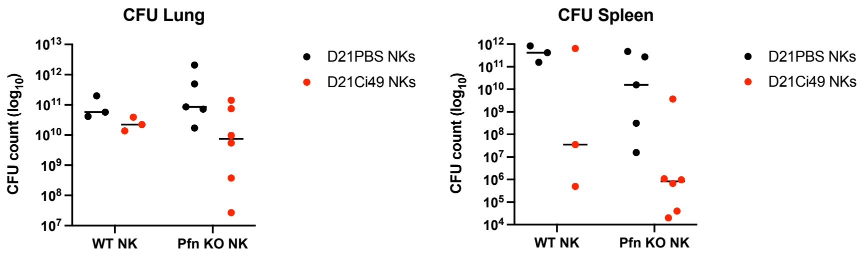

Figure R2: Bacterial counts at 40h post-infection in the lungs and spleen of mice having received either D21PBS NKs (black symbols) or D21SPN NKs (red symbols) from either WT mice or Perforin KO mice. Box plots where each dot represents an individual mouse, lines are the mean, error bars show min to max and dotted lines represent limit of detection.

Reviewer #3:

1. The infection with SPN is intranasal, and the target organ for SPN is the lung. However, the authors used splenic NK cells for adoptive transfer experiments. Could similar results be obtained with NK cells from other organs, in particular lung?
We agree with the reviewer that this is an important point. We have performed new experiments transferring purified Memory and Naïve NK cells from lung in vivo. The results are included in a new panel of figure 4 (Figure 4F), which shows Memory NK from lung contribute to reduce the numbers of CFUs recipient mice and shows that that lung NK cells from 21d post-infection also provide a protective effect to recipient mice.

2. Fig. 1: Do NK cells also respond to SPN + IL-15 alone (no IL-18)? How important is an inflammatory environment, mimicked by IL-18, for the NK cell response in presence of SPN?
We have performed a pilot experiment (see Figure R3) in which we have stimulated NK cells with IL15+SPN alone. The results show that, NK cells are not as activated (measured by GzmB+ cells) in the absence of IL18, and although an increased activation trend might be present in memory cells, the activation is more efficient and pronounced when both IL18 and IL15 are present during stimulation with SPN.

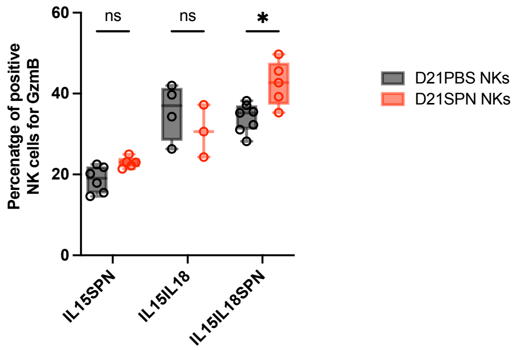

Figure R3: NK cells were highly purified from spleens of C57BL/6 mice (98% of purity) and stimulated *in vitro* with cytokines (IL-15 at 2 ng/ml, IL-18 at 1,5 ng/ml), and formaldehyde inactivated bacteria (MOI 20) for 24 hours. Percentage of Granzyme B$^+$ NK cells. Box plots where each dot represents a pool of mice from one experiment, lines are median, error bar show min to max. Data are representative of one experiment with n ≥ 4 pooled mice/group and n ≥ 3 experimental replicates/group.

3. Fig. 1: Do NK cells respond with a similar response if treated with another formaldehyde-inactivated bacterium?

These data were included in figure 6A and 6B, where we show that although the addition of inactivated SPN stimulated Granzyme B and Perforin specifically in memory NK cells, the incubation with inactivated L. monocytogenes or Streptococcus agalactiae (GBS) did not induce an increase of Granzyme B and Perforin (Figure 6A-B). These data therefore support the specificity of the response.

4. Fig. 1: How do NK cells respond to live, noninactivated SPN?
This is an interesting point to which we have not found a technical way to address it. Due to bacterial growth over the incubation time, we are unable to incubate NK cells with live bacteria for as long, as we do with inactivated bacteria (24h). The maximum possible incubation time with live bacteria is approximately 6 hours, at which point NK

cells are not yet activated and bacteria overtake the culture. Therefore, we cannot incubate the cells and the bacteria long enough to maintain the NK cells viable and see an effect in their activation.

5. SPN-mediated activation of myeloid cells has been shown to be TLR2- and TLR4-mediated. The authors should identify whether this is also true for memory NK cells. Is the recall response by NK cells altered if the respective receptors are blocked or knocked out?
   As TLR KO mice are extremely sensitive to infection, we do not believe it would be easily feasible to induce memory in these animals. We have however addressed this point in vitro. We have added new experiments in the manuscript where we have stimulated D21SPN and D21PBS NK cells in vitro with LPS or Pam3CSK4 (TLR4 and TLR1/2 agonists respectively), in parallel to SPN. We hypothesized that if memory NK cells are generated and activated in a TLR mediated manner, they would have an increased response when stimulated by LPS or Pam3CSK4. While memory NK cells had increased levels of GzmB following SPN stimulation, we did not observe differences in D21SPN compared to D21PBS NK cells stimulated by LPS or Pam3CSK4 (new Figure 6C). This result suggests that memory mechanisms are not mediated by TLR receptors. As a control, we have followed NK cells response to LPS or the TLR agonist by measuring Ifng positive cells (new Supplementary Figure 5B).

6. The authors suggest that the memory NK cell response is specific to the first pathogen the mice have been infected with (SPN). It would be highly relevant to identify the reason for this specificity. Do NK cells depend the on the same PRRs for responding to SPN and to L. monocytogenes? Furthermore, the main target organs for SPN and L. monocytogenes differ (lung vs liver). Can the same 'specific' NK cell memory response as e.g. in Fig. 1 be confirmed if mice were infected with L. monocytogenes instead of SPN and NK cells re-stimulated with the same pathogen? This would reveal important information and confirm the authors' statements concerning 'specificity' of the memory NK response.

   We appreciate the interest of the reviewer, and we agree on the interest of the points mentioned. However, to induce memory with L. monocytogenes would imply setting up a different infection method and dose, and we believe that this is beyond the scope of the paper. However, we have added experiments in the new manuscript to begin to address whether detection of bacteria is through PRRs. In these experiments we asked whether memory NK cells generated by infection with S. pneumoniae were reactive to other TLR agonists. This is not the case, as shown in the new figure 6C. Therefore, we believe that memory responses are not driven by activation of PRRs, consistent with in vivo findings of specificity.

7. The authors should extend the phenotypic characterization of the responding NK cells (vs non-responding NK cells), e.g. NK cell differentiation/maturation, Ki67. Since only bulk NK cells are compared, it may be that the differences are hidden when looking at this level which may be revealed when gating further down on NK cell subsets based on expression of Ly6C+, CD27low/neg, CD11b+ for example (Sun et al., Nature. 2009., Schuster et al., Immunity. 2023).

   We have investigated the phenotypic properties of responding vs non-responding NK cells by gating GzmB+ (responding) or Gzmb- (non-responding) in both Memory and Naïve NK cells stimulated in vitro with SPN. We have then measured the percentage of positive cells for CD11b and CD27. We have observed that cells with enhanced cytotoxicity (Gzmb+) have a more mature phenotype as the percentage of CD11b+ cells is significantly increased. This result is expected as previously published in Hayakawa et al., 2006, Chiossone et al., 2009 or Kim et al., 2002. In addition, we do not observe a difference in the percentage of CD11b+ NK cells between Memory and Naïve NK cells. We neither found significant differences in the percentage or MFI of CD27 in the analyzed conditions. This data are now presented in Supplementary Figure 5C.

8. Fig. S1C: A significant difference for the MFI of Ly49D is not visually clear from the data and difficult to believe – the authors should both increase the number of experiments and provide representative plots/histograms in order to confirm their statement.
   We believe that the difference on the MFI of Ly49D is not biologically significant. We provide here a zoom of the figure to appreciate the difference. In addition, at 21 days this difference is no longer present. We do not consider ethically valid to sacrifice more mice to repeat this control experiment.

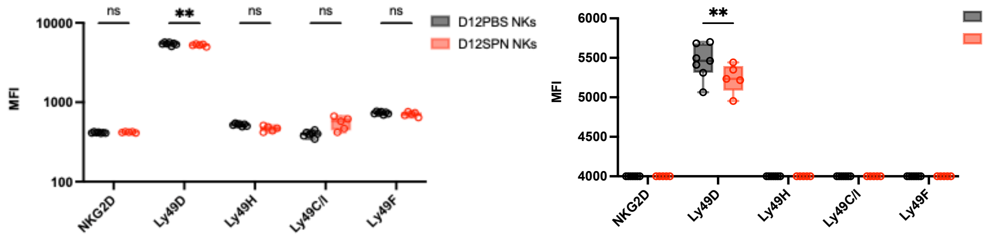

9. Fig. S2: The percentage of CD69+ NK cells in the lung seems overall rather low, and after 72h, it decreases even further. The authors should provide representative stainings for CD69 as well as data for CD69 expression before infection – does the frequency increase after 24h compared to before (mock-)infection, or is CD69 expression rather decreased for some reason at 72h and 21d? Furthermore, since higher percentages of CD69+ NK cells at 24h are even present in the PBS-control mice, it seems unlikely that this is an effect due to the infection. These data are confusing, and the authors must be careful with their conclusion of a low-level immune response. In relation to this, the authors should also reveal why granzyme B seems to increase in the control group (see also comment below).

We thank the reviewer for bringing up this point. We have carefully checked our data and realized that the values for CD69+ cells at 24h were incorrect. We have modified the graph with the right values. Now, it can be observed that at 24h and 72h there is an increase of CD69+ cells following the sub-lethal infection to induce memory. In the PBS condition, the percentage of CD69+ cells remain low at all timepoints and in the memory condition values are back to basal levels.

10. Fig. 2 and S2: From Fig. S2, no clear granzyme B signal is detectable in comparison to the isotype control reflecting that gzmb is not expressed to significant levels at 24, 72 and 21D (the few 'positive' events are rather likely an effect by spillover from other channels since I assume that this was not a FMO ctrl?). While it might be possible that lung NK cells express less granzyme B, splenic NK cells were found to also express granzyme B according to Fig. 2D, which is not supported by the representative data in Fig. S2D. In particular at 21d, Fig. 2D shows a clear percentage of granzyme B-positive cells in lung and spleen, which is not confirmed by the representative overlays in Fig. S2D. This is confusing, and the authors need to present more reliable data to support their statements concerning granzyme B expression. In relation to this, the authors also need to present representative data for perforin expression at the different timepoints and groups in order to support their statements.
    We thank the reviewer for the suggestions. We have now revised the gating of these experiments and added more replicates. Also, the representative data has been revised for GzmB levels at 24h, 72h and 21D, where the levels of GzmB are low in all cases and there is no difference between Naïve and Memory NK cells at any timepoint. In addition, as the reviewer suggests, we have added MFI and representative data for perforin.

11. It is unclear why only splenic NK cells have been analyzed in their phenotype (Fig. S2). The authors should add analyses on NK cells from other organs, in particular the lungs.
    Figure S2 is dedicated to the phenotypic characterization of the lung. We think the reviewer switched the two and is probably asking for phenotypic characterization of splenic NK cells. We are including below NK cell responses in spleen following sub-lethal infections (Figure R1). We do not observe any transient response in NK cells percentage, number or activation in the spleen following sub-lethal infection, consistent with the fact that we do not detect CFUs in this organ.

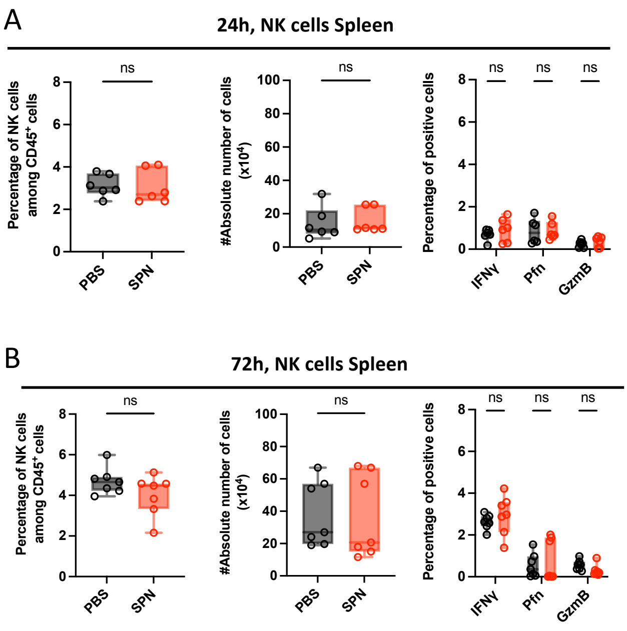

Figure R1: Organs were collected at 24h (**A**), 72h (**B**). Percentage of NK cells (NK1.1$^+$ CD3$^-$) among CD45$^+$ cells, percentage of IFNγ^+, Perforin$^+$, Granzyme B$^+$ NK cells in spleen (**right panel**). Box plots where each dot represents an individual mouse (black dots for uninfected mice, red dots for infected mice), lines are the median, error bar show min to max. Data are pooled from two with n ≥ 3 mice/group. ns, not significant. Mann-Whitney test for single comparisons and 2way ANOVA test for multiple comparisons.

12. Fig. 4A: It would be interesting to see whether the percentage of IFN-g+ NK cells further increase e.g. at 72h, or whether the peak of the response of D21SPN NK cells is reached earlier.
    We agree this would be interesting. Unfortunately, the proposed experiment is not technically feasible as following lethal infection, recipient mice need to be sacrificed at 48h and are too sick to carry out the experiment in later timepoints.

13. The number of experiments should be increased for several datasets throughout the manuscript. In general, more than just one experiment should be performed for each figure. It is also not entirely clear why the authors sometimes show the data as pooled experiments, and in other plots each individual mouse. This is confusing, and the paper would benefit from a more consistent data presentation.
    We have now increased the number of replicate experiments throughout the paper Specially in Figures 2, Supplementary 2 and Figure 6. For the in vitro experiments, purified NK cells from several mice are pooled together and distributed in wells for stimulation with different conditions, as this reduces the mouse to mouse variability.

14. The authors state that 'transferred congenic CD45.2+ NK cells were circulating and detectable at similar percentages in lungs and blood, suggesting there is no preferential trafficking between D21PBS and D21SPN NK cells'. As the lungs are perfused extensively with blood, any differences in NK cell number/percentages could be completely diluted and therefore missed. The lungs could be flushed to clear the blood and then stained to determine NK cell numbers/ percentages. Alternatively, fluorescently labelled anti-CD45 could be given IV before analysis to show what is actually circulating and what may be resident in the parenchyma instead. Again, subset specification may also pull out more interesting results.
    We thank the reviewer for the suggestion. However, as we do not specifically investigate the residency and trafficking of memory NK cells, we do not support

sacrificing more mice to test this in all our conditions. However, we will take this suggestion into consideration for future experiments.

15. Fig. 5D: The reduction of CXCL1 in the D21SPN NK cells indicates that this chemokine was e.g. consumed by cells infiltrating the lung, hence, this is not a clear indicator for the lack of NK cell infiltration. The authors should combine this analysis with the expression patterns of the respective chemokine receptors on NK cells.
    We thank the reviewer for the comment, it would be interesting to check this in a further study, however, we believe that it is outside of the scope of this paper.

16. Fig. 6: The authors here show a population of granzyme B+ as well as perforin+ NK cells. Does the phenotype differ to the respective negative NK population?
    We have investigated the phenotypic properties of responding vs non-responding NK cells by gating GzmB+ (responding) or Gzmb- (non-responding) in both Memory and Naïve NK cells stimulated in vitro with SPN. We have then measured the percentage of positive cells for CD11b and CD27. We have observed that cells with enhanced cytotoxicity (Gzmb+) have a more mature phenotype as the percentage of Cd11b+ cells is significantly increased. This result is expected as previously published in Hayakawa et al., 2006, Chiossone et al., 2009 or Kim et al., 2002. In addition, we do not observe a difference in the percentage of Cd11b+ NK cells between Memory and Naïve NK cells. We also found no significant differences in the percentage or MFI of CD27 in the analyzed conditions. This data is now presented in Supplementary Figure 5C.

Part III – Minor Issues: Editorial and Data Presentation Modifications
Please use this section for editorial suggestions as well as relatively minor modifications of existing data that would enhance clarity.

We can see all of this once we have addressed the major points as many of them might go away or be changed anyway.

Reviewer #1:

1. Fig 2B appears to be from x1 experiment. Are these data representative from at least 2 repeats? If not, they should be repeated.
   We have now added replicates to all experiments and all data represented in Figure 2 is coming from at least 2 experiments.

2. In figure legends (ex, Fig 1) it is unclear what "each dot represents a pool of mice" refers to. Is this meant to be pool of cells from one mouse?
   We have now modified the Figure legends clarifying that “D21PBS NK cells and D21SPN NK cells are purified and pooled from n ≥ 4 mice/group and incubated in n ≥ 3 experimental replicates/group. Box plots where each dot represents an experimental replicate (black dots for D21PBS NK cells, red dots for D21SPN NKs cells)”

3. Why is there high baseline granzyme B detected for 21 day NK cells (Fig 2D) vs other panels?
We have now revised the gating of these experiments and added more replicates. Also, the representative data has been revised for GzmB levels at 24h, 72h and 21D, where the levels of GzmB are low in all cases and there is no difference between Naïve and Memory NK cells at any timepoint. In addition, we have added MFI and representative data for perforin.

4. Fig 3B update legend to include 24 h.
We thank the reviewer for the observation, it has been added.

5. Discussion pg 9, Fig 4C results update to reflect that there are reduced burdens in IFNGR-/- mice (line 214), don't see significantly reduced lung bacteria (line 217).

   We have modified the sentence to: "Importantly, we still found a significant reduction in bacterial numbers in the spleen, and a similar trend in lungs, of Ifngr KO recipient mice having received WT D21SPN NK cells compared to Ifngr KO recipient mice having received WT D21PBS NK cells (Figure 4C)".

6. Methods are missing for GBS growth.
We thank the reviewer for the observation, it has been added.

Reviewer #2:

1. Pneumococcus is referred to throughout the manuscript as an extracellular pathogen. This is surely its primary lifestyle, but many studies have shown the ability of pneumococci to access intracellular compartments and demonstrated that intracellularity, whilst rare, can make important contributions to infection outcomes (see https://pubmed.ncbi.nlm.nih.gov/29662129/, https://pubmed.ncbi.nlm.nih.gov/33216805/). Some discussion could be added, regarding the possibility that NK cell memory might be mediating protection via targeting intracellular subpopulations of bacteria.
We have now added to the discussion lines 428-432: "Although the pneumococcus lifestyle is primarily extracellular, a few studies have reported occasional intracellular replication within splenic macrophages and lung epithelial cells (Ercoli et al. 2018; Badgujar et al. 2020). In our *in vivo* studies, we therefore cannot exclude the possibility that memory NK cells are protecting the host by targeting cells infected with intracellular pneumococci".

2. The authors describe bacterial sensing by NK cells as the mechanism behind the memory phenotype they observe. Indeed, the data they present supports some contribution from direct sensing mechanisms, but the possibility remains that the original cue for memory NK cell responses to develop in vivo requires other immune cells. Previous studies have suggested that inflammasome-driven macrophage responses can promote NK cell memory

(https://pubmed.ncbi.nlm.nih.gov/27287410/), whilst others have shown the pneumococcal infection drives inflammasome responses that ultimately lead to an NK cell IFNg response in the lung (https://pubmed.ncbi.nlm.nih.gov/21085613/). Some discussion of these points in the context of the authors' own findings would be welcome.

We have now added to the discussion lines 387-392: We hypothesize that NK cells acquire memory by a direct interaction with pneumococcus, but we cannot rule out the possibility that other immune cells are involved in this process. As in other NK cell memory models (van den Boorn et al. 2016; Venkatasubramanian et al. 2017; Sun et al. 2012; Rölle et al. 2014), some pro-inflammatory cytokines and costimulatory molecules expressed by other cells might be required for generating *S. pneumoniae* memory NK cells.”.

3. A little extra clarity on the murine models is needed in places. What is the rationale behind the consecutive dosing, over two days, when performing infections? Does NK cell memory require this double dose?

   We have now added in lines 100-102 “Infecting mice with two consecutive doses over two days improved reproducibility of our results compared with one dose, suggesting robust colonization is important.”.

4. In Figure 3, was the dose 1x10^7 (line 167) or 5x10^6 (line 174)?
   We thank the reviewer for the observation, the dose of infection used every study has been clarified.

5. Line 112: Replace 'than' with 'as'.
   We thank the reviewer for the observation, it has been changed.

Reviewer #3:

1. The response of NK cells is dependent on the SPN-serotype. Why did the authors select serotype 4 for their study, and is NK cell memory function detectable with other SPN serotypes? The authors should discuss this.
   In the methods section, we have added a clarification on the serotype used for the study, a commonly used pathogenic serotype of SPN. Studies with other serotypes are beyond the scope of this study.

2. The authors should mention that the results are derived from a mouse model in the abstract.
   As suggested by the reviewer, we have added this information in the abstract.

3. When the authors write e.g. 2.105, I assume they mean 2x105? Please revise throughout the manuscript.

We thank the reviewer for the observation, it has been changed.

4. In line 158 the authors state that after 21 days post infection that immune cells are similar to uninfected controls 'both in their number and activity'. No activity was measured. The authors should modify this sentence.
   We have replaced activity by "level of activation".

5. The authors repeatedly state that they isolate or stimulate 'highly purified memory NK cells', which is not the case. They are purifying bulk NK cells which probably contain memory NK cells to differing degrees. Therefore, although the NK cell population may be highly pure (they state >98% purity), this does not mean they are purifying memory cells. Purifying memory cells suggests they are identifying a distinct NK population and they are not. The authors should revise these statements.
   We have modified "we purified naïve or memory NK cells" for "we purified NK cells from naïve or previously infected mice", to clarify this point.

6. In Fig. 5C and S4B, only a handful of innate cells are described. This is not exhaustive and does not include any adaptive cells, therefore their conclusion stating 'protection provided by memory NK cells is not through enhanced recruitment or activation of inflammatory cells' is over-interpreted. This sentence should be modified. For example, NK cells are known to recruit CD8+ T cells which were not quantified, neither were inflammatory monocytes which are common inflammatory cells recruited upon infection.
   T cells were part of the immune cells tested (see Figure 5). We have now modified the sentence to "These results suggest that the protection provided by memory NK cells is not through enhanced recruitment or activation of any of the inflammatory cells tested".
